# Cross-Disciplinary Research on Learning and Instruction – Coming to Terms

**DOI:** 10.3389/fpsyg.2021.562658

**Published:** 2021-05-11

**Authors:** Nicole Heitzmann, Ansgar Opitz, Matthias Stadler, Daniel Sommerhoff, Maximilian C. Fink, Andreas Obersteiner, Ralf Schmidmaier, Birgit J. Neuhaus, Stefan Ufer, Tina Seidel, Martin R. Fischer, Frank Fischer

**Affiliations:** ^1^Department of Psychology, LMU Munich, Munich, Germany; ^2^Department of Mathematics Education, Leibniz Institute for Science and Mathematics Education, Kiel, Germany; ^3^Institute of Medical Education, University Hospital, LMU Munich, Munich, Germany; ^4^TUM School of Education, Technical University of Munich, Munich, Germany; ^5^LMU University Hospital, Medizinische Klinik und Poliklinik IV, Munich, Germany; ^6^Biology Education, Faculty of Biology, LMU Munich, Munich, Germany; ^7^Mathematics Education, LMU Munich, Munich, Germany

**Keywords:** conceptualization, cross-disciplinary research, collaborative problem solving, transdisciplinary research, interdisciplinary research, joint theoretical framework, joint methodological approach

## Cross-Disciplinary Research Collaborations

Research in universities and other organizations is often conducted within established disciplines that are historically based and highly arbitrary (Campbell, 2014). However, emergent phenomena fail to fit into disciplinary boundaries, making cross-disciplinary research necessary, often involving corresponding collaboration (Hall et al., 2008).

One area of research involving complex phenomena that cannot be well addressed by one discipline alone is learning and instruction in higher education. Higher education programs aim to teach professional knowledge to students as a prerequisite for their later professional activities (Blömeke et al., 2015). For example, in teacher education programs usually focus on content knowledge (CK), pedagogical content knowledge (PCK), and pedagogical-psychological knowledge (PK) (see Shulman, 1987). In order to teach such knowledge, it seems reasonable and is increasingly common that psychologists and educational scientists, in addition to experts in the subject matter domains, are involved in designing study programs. Similarly, it also seems reasonable to involve researchers from these various domains for conducting research on how to facilitate teaching in higher education programs. Thus, cross-disciplinary collaboration is the rule rather than the exception in higher education *practice* and is becoming increasingly common in *research* on higher education. An example for a cross-disciplinary research endeavor in learning and instruction is a research unit on facilitating diagnostic competences in simulation-based learning environments in the university context in which researchers from subject matter domains (biology education, mathematics education, and medical education) are working together with researchers from education and from educational psychology^1^.

Even though there is a decent amount of research on cross-disciplinarity, for example from the science of team science (Hall et al., 2018, 2019), there is only limited research on cross-disciplinarity in the field of learning and instruction, and especially on collaborative processes. In this opinion article, we claim that ideas and concepts from the field of collaborative problem solving have the potential to yield valuable insights when designing or conducting cross-disciplinary research in learning and instruction.

## Conceptualization of Cross-Disciplinary Research Endeavors

There is substantial evidence on some specific features that positively influence cross-disciplinary research collaborations, such as team formation, team composition, or institutional factors (e.g., Epstein, 2014; O’Donnell and Derry, 2014; Hall et al., 2018, 2019). However, it remains unclear how prerequisites such as the intended form of the cross-disciplinary collaboration influence the collaborative problem-solving process, and second, how the collaborative problem-solving process itself influences and is influenced by other factors such as aspects of the cross-disciplinary team or the production of joint artifacts.

**FIGURE 1:**
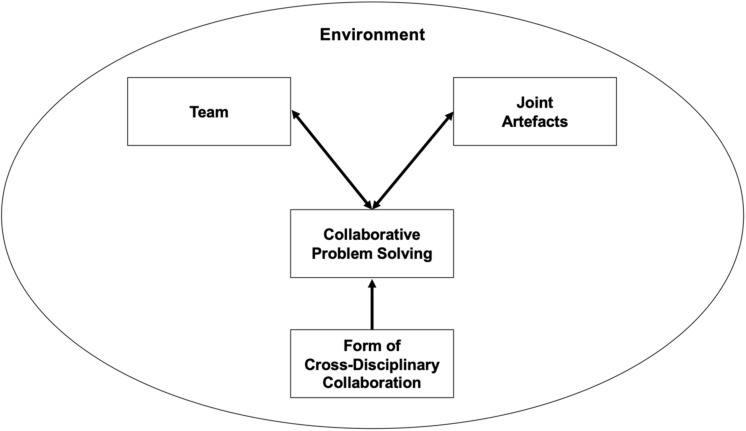
Conceptualization of cross-disciplinary research in learning and instruction.

We introduce a conceptualization of how ideas and concepts from the field of collaborative problem solving are useful to address challenges that arise from cross-disciplinary research (see Figure 1). The conceptualization is based on existing approaches to cross-disciplinary research (e.g., Epstein, 2014; O’Donnell and Derry, 2014; Hall et al., 2018, 2019) and extends these approaches by introducing processes and skills from collaborative problem solving (Hao and Mislevy, 2019; Hao et al., 2019).

The basis of our conceptualization are the three different forms of cross-disciplinary research that are commonly differentiated: multidisciplinary, interdisciplinary, and transdisciplinary (e.g., Lattuca, 2003; Slatin et al., 2004; Collin, 2009; Hall et al., 2012; Klein, 2017). Which form of cross-disciplinary research is intended, can have an influence on the collaborative problem-solving process in the way that it sets the stage for which collaborative problem-solving skills are of major importance. Collaborative problem solving builds the core of our conceptualization. We discuss how factors of the cross-disciplinary team reciprocally influence the processes of collaborative problem solving and how the collaborative problem-solving process itself and the development of joint artifacts influence each other. The environment, in which a cross-disciplinary research endeavor takes place, surrounds the other elements of the conceptualization building another important factor to consider in cross-disciplinary research in learning and instruction.

### Form of Cross-Disciplinary Collaboration

Forms of cross-disciplinary collaboration differ in their collaborative problem-solving process and build thus the basis for the conceptualization. Three forms that are commonly differentiated are *multidisciplinary* research, *interdisciplinary* research, and *transdisciplinary* research (e.g., Lattuca, 2003; Slatin et al., 2004; Collin, 2009; Hall et al., 2012; Klein, 2017). However, so far there is no agreed upon definition for each form (e.g., Hall et al., 2008). For the purpose of our analysis, we use the following differentiations (Klein, 2017): In *multidisciplinary* research, different disciplines work on different aspects of a problem independently within their disciplinary boundaries. Researchers from different disciplines contribute specific knowledge and skills with the goal to address a certain phenomenon or issue from multiple perspectives. In *interdisciplinary* research, existing disciplinary approaches are restructured and integrated in order to address a problem relevant for all participating disciplines. Interdisciplinary research can be seen as a spectrum reaching from researchers borrowing concepts and methods from other disciplines to answer a specific research question up to the development of new frameworks that are valid across disciplines (Pohl et al., 2021). Researchers share their knowledge and then identify which concepts or methods from the other disciplines are necessary for answering research questions within their own discipline or that go beyond their own disciplinary boundaries. In interdisciplinary teams, researchers’ still focus on their own disciplines even though disciplinary boundaries are crossed to some degree to make the points of contact between the disciplines compatible (Choi and Pak, 2006). *Transdisciplinary* research also seeks to integrate different lines of work from contributing disciplines (Klein, 2010; Pohl, 2010). A key aspect of transdisciplinary research is the collaborative co-production of knowledge from researchers from different disciplines, and possibly also stakeholders from private or public sectors with the goal to solve societal problems (Pohl et al., 2021). Whereas in interdisciplinary research actions in the collaborative process are described with linking, blending, fusing, and synthesizing, actions in transdisciplinary research are transcending, transgression, and transforming (Klein, 2010). Disciplinary boundaries can be challenged on purpose in the process of transdisciplinary research (Pohl et al., 2021). Whereas the current discourse on cross-disciplinary research distinguishes between three discrete forms (multidisciplinary, interdisciplinary, and transdisciplinary), there are considerations that place them on a continuum (Mennes, 2020).

### Collaborative Problem Solving

We want to make the claim that even though cross-disciplinary research in learning and instruction can be considered through the lens of collaborative problem solving, the intended form of cross-disciplinary collaboration can influence the role that collaborative problem solving plays in that process. Main aspects of collaborative problem solving important for cross-disciplinary research are collaborative problem-solving skills and different roles to help stimulate the problem-solving process.

Collaborative problem solving involves cognitive skills, such as defining the problem at hand and social skills, such as establishing a shared understanding (Graesser et al., 2018). Regarding the collaborative problem-solving process, four skills are considered to be of major relevance (Liu et al., 2016; Hao and Mislevy, 2019): (1) *Sharing ideas* refers to how individuals bring divergent ideas into a collaborative process (Liu et al., 2016). (2) *Negotiating ideas* refers to building collaborative knowledge and constructing processes within a group. Negotiating occurs by comparing alternative ideas and their associated evidence. Subprocesses of negotiating ideas include agreeing, disagreeing, requesting clarification, elaborating on each other’s ideas, and identifying gaps (Liu et al., 2016). Collaborative team knowledge is produced in this process (Liu et al., 2016). (3) *Regulating problem-solving activities* is a social skill that refers to the coordination of discourse within a team. An example is to highlight the goal of a discussion, such as finding an up-to-date instrument to measure motivation. An important aspect regarding the regulation of problem-solving activities is that members’ individual ideas about what collaboration looks like might differ more in cross-disciplinary projects than in mono-disciplinary projects. External guidance might be needed to ensure successful collaboration (von Wehrden et al., 2019). (4) The social skill of *maintaining conversation* refers to communication that is not directly topic-related but maintains a positive atmosphere (Liu et al., 2016). This kind of non-topic-related communication seems to be of major importance in cross-disciplinary teams in order to support the collective communication competence of the team (Thompson, 2009). Research on cross-disciplinary research collaborations from other fields suggests examining how the involved disciplines differ in their way of collaborative problem solving and communicating and then providing enough guidance while still offering enough possibilities for participation in all collaborative problem-solving processes (König et al., 2013).

Depending on the form of cross-disciplinary collaboration, different collaborative problem-solving skills seem to be central. In a cross-disciplinary research unit in learning and instruction, regulating the problem-solving process is central for multidisciplinary goals. This importance is based on the fundamentally different perspectives on the same problem by researchers from different disciplines, e.g., subject matter didactics, educational psychology, and educational science. In addition to the need to regulate problem-solving processes within the team externally, coordinating resources that exist in the different disciplines and defining interfaces might be necessary. For example, it might be important to organize and moderate meetings in which different disciplinary perspectives on a joint problem can be juxtaposed. For interdisciplinary goals, sharing knowledge across disciplines seems particularly important in addition to regulating the process (see Liu et al., 2016). For interdisciplinary and transdisciplinary goals, negotiating can be considered a specifically important skill for grounding and finding a shared language across disciplines (Bromme, 2000). Based on these examples, we hypothesize that each form of cross-disciplinary collaboration (multidisciplinary, interdisciplinary, and transdisciplinary) requires unique collaborative problem solving and communication skills, because they differ in their main goals as well as in the means of achieving and communicating these goals.

Possibly, it can be beneficial for the definition of specific working routines, such as for the development of learning environments, to assign different collaborative problem-solving activities to different roles. Roles can be conceptualized with reference to internal collaboration scripts. Internal collaboration scripts are mental schemas that typically include a set of roles and associated activities (Fischer et al., 2013). These internal scripts may differ widely across disciplines. For example, the collaboration script in one discipline can involve that junior researchers first formulate a draft for a manuscript and later senior researchers comment on that draft. In other disciplines, junior researchers might be involved at other stages of the publication process. Therefore, making the task of specific roles explicit during interactions within the team seems important.

The regulation of the problem-solving process should be assigned to the role of a facilitator who mediates between actors from different disciplines (see also Bammer, 2016; Salazar et al., 2019). The facilitator can take over processual leadership tasks to ensure that the interactions between team members are productive (Gray, 2008). In order to support the development of joint artifacts, it seems reasonable to spend resources on a facilitator with their own research experience at least on the post-doc level.

### Team

When building a cross-disciplinary research team, the science of team science has already described important aspects for team composition and team formation (e.g., Hall et al., 2018, 2019). We focus on aspects of collaboration that are in close connection to collaborative problem solving. These aspects include overlapping expertise within the team, a strategy for publications, and a clear shared goal.

A deep understanding of more than one discipline is difficult to achieve (Pohl and Hadorn, 2008). Most research teams have to engage in collaborative problem solving between various researchers with deep discipline specific knowledge. Campbell (2014) uses the metaphor of a fish’s scales to describe the composition of successful cross-disciplinary teams. In his model, each fish scale symbolizes one individual with a unique set of expertise. In order to build a successful team, each “fish scale” has to overlap to a certain degree with the neighboring fish scales. There are fish scales that are close to each other and others that are further apart. Those further apart from each other are not directly connected but are indirectly connected via the other fish scales. What can be drawn from Campbell’s (2014) metaphor is that it is not necessary that researchers from all disciplines collaborate directly in a collaborative problem solving process, which would be highly laborious; rather, they may also be connected via researchers from other disciplines.

In research on learning and instruction it seems likely that the “connecting fish scale” is represented by researchers from the educational sciences or educational psychology because these disciplines are concerned with learning in general. For example, in the research unit on facilitating diagnostic competences in simulation-based learning environments researchers from mathematics education and medical education did not have a direct link at first. These two groups of researchers were only indirectly connected via their collaboration with the field of psychology. It seems possible that researchers from the connecting fish scale can have a major influence on the collaborative problem-solving process because they play a major role in regulating the problem-solving process.

A major challenge of cross-disciplinary teams is the lack of an adequate joint reward system during the collaborative problem solving process (O’Donnell and Derry, 2014). Within disciplinary boundaries it is relatively clear how much a publication in a journal, book, or conference proceedings will benefit a researcher’s career. For example, publications in conference proceedings are typically less valued than international journal publications for an educational psychologist. However, the value of a publication becomes less clear when it appears outside of a researcher’s disciplinary boundaries or in an interdisciplinary journal. Furthermore, joint publications face additional problems such as over-inclusive authorship (Elliott et al., 2017; Settles et al., 2018) or what disciplines see as reliable epistemic processes or epistemic ideals (Chinn et al., 2011). The entire meaning of collaboration in a team of authors varies across disciplines. An exclusive focus on cross-disciplinary publications may be particularly problematic for young researchers, whose goal is to develop a record and profile of expertise *within* their disciplinary field. It seems even reasonable to suggest that young researchers should be encouraged to submit their first manuscripts primarily to disciplinary journals.

For cross-disciplinary research in learning and instruction, it is a major challenge to identify phenomena and questions that allow for research that is relevant or even cutting edge in all of the participating disciplines (e.g., Epstein, 2014). Examples of participating disciplines in learning and instruction are psychology, education, and various subject matter didactics such as mathematics education or biology education. In order to have interdisciplinary and transdisciplinary goals in a research endeavor in learning and instruction, it seems crucial to identify a phenomenon that makes integration of concepts and methods from different disciplines necessary. A helpful method for defining such goals may be integrating question that bring together different avenues of inquiry (Cosens et al., 2011).

### Joint Artifacts

Another major aspect for cross-disciplinary research in relation with collaborative problem solving is the development of joint artifacts. O’Donnell and Derry (2014) stress the importance of artifacts, which they call *tools*. For research on learning and instruction in higher education it seems characteristic that different concepts, methods, and technologies are used in the subject matter domains (e.g., biology or mathematics), in psychology, and in educational science. Therefore, it seems reasonable to suggest the development of three types of joint artifacts early in the collaborative problem-solving process in order to identify possible barriers but also potentials for innovation: a joint conceptual framework, a joint methodological framework, and a joint technological framework. In order to develop such artifacts it seems advisable to include an overarching coordination mechanism that ensures methodological and conceptual standardization and progress (see König et al., 2013). The development of joint artifacts can be of major relevance for collaborative problem-solving processes, such as information sharing and negotiating.

• A joint conceptual framework can identify relevant theoretical ideas and their interconnections. It can ensure that common ground exists and that terms are defined precisely.
• A joint methodological framework refers to methods and more detailed research practices. A precise description of methods is important because methods and best practices vary between disciplines. What is considered a gold standard in one discipline can be seen as less important in another discipline; for example, an empirical-experimental approach is difficult to combine with hermeneutic methods.
• A joint technological framework defines the technology relevant for collaboration and for addressing the research questions. Every discipline in the context of learning and instruction has its own set of preferred research technologies, for example simulations that create extensive logfiles to measure and facilitate learning (Fink et al., 2020). Joint technologies may help to integrate data from different research projects, and later transfer the results into practice. In order to have a suitable technology for learning, it can be necessary for researchers to develop their own software.

### Environment

The last aspect in our conceptualization of cross-disciplinary research in learning and instruction is the environment that surrounds the other aspects. In connection with cross-disciplinary collaborations there are various environmental factors such as societal and political factors that influence whether a research endeavor will receive attention and funding. In this section we focus on a factor that researchers can influence to a certain degree: the institutional climate.

The institutional climate refers to the perceptions, attitudes, and expectations of an institution toward cross-disciplinary research. Epstein (2014) argues that the institutional climate can support horizontal, cross-disciplinary structures that allow researchers to cluster around phenomena. As the institutional climate in many academic institution may only change slowly and gradually, it can take years of preparation and the completion of smaller projects to develop a sound environment for a research collaboration. In particular, it may only marginally be susceptible to individual members of the institution, making joint efforts and initiatives necessary. Thus, it seems reasonable to plan enough time for preparing both capacity as well as the environment for the actual research endeavor. It seems advisable to start with a smaller-scale project, such as the joint supervision of a single Ph.D. project or a joint publication. A well prepared institutional climate might also be beneficial for collaborative problem solving and particularly for maintaining conversation.

## Discussion

Cross-disciplinary research collaborations in the context of learning and instruction are of critical importance to address the complex problems of 21st century education. However, many promising projects fail beyond the actual research conducted due to avoidable issues (Fam and O’Rourke, 2021). The research reviewed here allows for formulating reasonable hypotheses about favorable processes and conditions with a psychological focus from the perspective of collaborative problem solving. These hypotheses may support scientific achievements such as the use of pilot projects, the early development of joint artifacts, conceptual, methodological, and technical frameworks, or the role of an experienced facilitator supporting the collaborative problem-solving process through intellectual grounding, coordination and negotiation. Whether and under which conditions these hypotheses are valid for cross-disciplinary research collaborations on learning and instruction and beyond remains an open empirical question. In further research the theoretical foundation as well as the relationship between the four aspects of our proposed conceptualization should be further expanded and specified using theories on science and technology studies (e.g., Hackett et al., 2008), actor-network theory (e.g., Latour, 1996), or theories on complex systems (e.g., Stacey, 1995). We believe our proposed conceptualization based on theoretical considerations and on our own experiences in a cross-disciplinary research unit on facilitating diagnostic competence in simulation-based learning environments can provide helpful terminology and some theory-inspired heuristics on how to realize the great potentials and to avoid the stumbling blocks when attempting the challenging task of cross-disciplinary research collaboration in learning and instruction.

## Author Contributions

All authors listed have made a substantial, direct and intellectual contribution to the work, and approved it for publication.

## Conflict of Interest

The authors declare that the research was conducted in the absence of any commercial or financial relationships that could be construed as a potential conflict of interest.

## Footnotes

1 COSIMA website: https://www.for2385.lmu.de
